# Online assessment of narrative macrostructure in adult Irish-English multilinguals

**DOI:** 10.3389/fpsyg.2022.916214

**Published:** 2022-07-26

**Authors:** Stanislava Antonijevic, Sarah Colleran, Clodagh Kerr, Treasa Ní Mhíocháin

**Affiliations:** Discipline of Speech and Language Therapy, School of Health Sciences, National University of Ireland Galway, Galway, Ireland

**Keywords:** macrostructure, narrative, story grammar, Irish, Multilingual Assessment Instrument for Narratives MAIN, narrative production, narrative comprehension, internal state terms

## Abstract

**Background:**

Online assessment of narrative production and comprehension became an important component of language assessment during the COVID-19 pandemic. This study aimed to establish quantitative measures of narrative macrostructure in the production and comprehension of adult Irish-English bilinguals in an online assessment.

**Methods:**

A total of 30 Irish-English bilingual adults participated in an online assessment of oral narrative production and comprehension. Narratives were elicited using LITMUS-MAIN for Irish and English. Story-tell elicitation method was used for all stories. Twenty participants produced Baby Birds and Baby Goats story pairs while 10 participants produced Cat and Dog story pairs. Quantitative measures of story structure, comprehension score, and the overall number of Internal State Terms (ISTs) in production and comprehension were compared across the story pairs, languages, and the output type (production vs. comprehension).

**Results:**

A general linear model indicated no differences in either story structure or story comprehension scores across languages for both sets of stories. Combined analysis for all participants and stories indicated no difference in the story structure scores or comprehension scores across the languages or the story pairs. While the overall number of ISTs was the same across languages, a higher number of ISTs was observed in comprehension relative to production in both languages for Cat and Dog story pair only, but not for Baby birds and Baby goats' stories. The major benefit of using online assessment was the accessibility of participants. The major drawback was the inability to control the environment and the quality of the internet connection.

**Conclusion and implications:**

While online assessment increased the availability of participants, which is a significant factor in rural Ireland characterized by low population density and the high percentage of Irish speakers, the availability of stable internet connection limited the applicability of online assessment. Measures of narrative macrostructure were stable across the languages and the story pairs. This is important because of high variability in exposure to Irish, frequent code-switching, and a high number of morphosyntactic errors due to rapid language change that characterizes Irish-English bilinguals. Identifying reliable measures of language performance for Irish-English adult speakers is an important step toward establishing developmental norms for Irish-English bilinguals.

## Introduction

While online language assessment has been present in speech and language therapy/pathology for a while, telepractice came into focus recently due to the interruption of in-person assessments at the start of the COVID-19 pandemic. The restrictions in conducting in-person speech and language assessments created an urgent need to validate online assessments and assessment protocols. Based on several studies that compared speech and language assessments online and in-person, Peña and Sutherland (2022) concluded that it is possible to reliably assess children using online procedures. Especially relevant here is the study by Pratt et al. (2022) who compared online and in-person narrative comprehension using the Multilingual Assessment Instrument for Narratives (MAIN; Gagraina et al., 2019) in English and Spanish. The study indicated a high correlation between the scores reflecting comprehension of story macrostructure for online and in-person assessments. This is an encouraging finding and calls for further research into using the MAIN (Gagarina et al., 2019) as an online assessment of narrative production and comprehension.

The current study used the MAIN in Irish (O Malley, 2019) and English (Gagarina et al., 2012, 2019) to assess narrative production and comprehension in Irish-English multilingual adults. Irish (*Gaeilge*) is one of the three official languages but at the same time a minority language of the Republic of Ireland. During the last 30 years, a significant decline has been noted in the use of Irish in the homes and wider communities resulting in almost universal bilingualism with English. Rapid language change is a consequence of the close contact with English and includes changes in the use of phonology, morphology, and syntax; increased use of direct translations from English; and frequent code-switching with English (Ó Catháin, 2016; Nic Fhlannchadha and Hickey, 2019, 2021). Due to the rapid language change, it can be challenging to judge grammatical accuracy and decide what are acceptable morphosyntactic forms in the current use of Irish. This further constraints the use of language assessments focusing on morphosyntax such as sentence repetition tasks or assessment of narrative microstructure (Antonijevic et al., 2017, 2020). Instead, a narrative assessment focusing on macrostructure can be a more ecologically valid and more reliable language assessment for Irish. To support language assessment through Irish in speech and language therapy/pathology, the MAIN (Gagarina et al., 2019) was adapted to Irish (O Malley, 2019).

Narratives offer a less biased method of assessing language in bi/multilinguals than norm-referenced, standardized assessments because their structural aspects are shared across languages (Paradis et al., 2010; Peña et al., 2014; Boerma et al., 2016). In addition, narratives include the interpretation of knowledge beyond the specifics of a particular language (Gagarina et al., 2012). Narratives can be analyzed at the levels of microstructure and macrostructure (Gagarina et al., 2015). The microstructure is specific to individual languages as it refers to the lexical and grammatical elements used to form coherent narratives (Boerma et al., 2016; Bohnacker, 2016). Macrostructure refers to the global organization of the story that is fairly similar across languages (Gagarina et al., 2019). The current study focuses on the MAIN (Gagarina et al., 2012, 2015, 2019), a narrative assessment that was specifically developed for multilingual children from diverse linguistic and cultural backgrounds as one of the assessments in the LITMUS battery created within the Cost Action IS0804 “Language Impairment in Multilingual Society: Linguistic Patterns and the Road to Assessment.” The original hypothesis of the group was that the story grammar knowledge as reflected in the narrative macrostructure would be invariant across languages of multilinguals (Gagarina et al., 2015). The MAIN includes four parallel stories comparable in the storyline, characters, and the number and structure of the episodes. The macrostructure of each story includes three full episodes depicted across six pictures. Episodes contain three core components: Goal (G), the objective of the main character; Attempt (A), their action aimed at achieving the goal; and Outcome (O), the result of the action. Episodes are framed with two Internal State Terms (ISTs). The initiating IST refers to the main character's emotional or cognitive state that initiates the setting of the goal and the attempt to achieve the goal. Closing IST is a reaction to the outcome of the action aimed at achieving the goal. Therefore, the structure of a full episode can be represented as IST_(initiating)_-GAO-IST_(reaction)_. The important difference between these different structural components is that characters' actions and the outcomes of those actions are explicitly depicted while their goals and internal states need to be inferred by the narrator from the elicitation pictures (Gagarina et al., 2012, 2015, 2019). The same structure in all four stories enables comparison of the assessment scores across languages and different elicitation modes (tell, retell and tell after a model story). It also allows for pre- and post-assessment without the risk of training effects (Pesco and Kay-Raining Bird, 2016), and ensures that any differences in language performance are not caused by variations in task difficulty (Kapalková et al., 2016).

Comprehension of narrative macrostructure is assessed through ten questions referring to the Goal of the main character, their Attempt to achieve the goal, the Outcome, and the two ISTs of each episode. Questions related to ISTs provide information on the child's metalinguistic and metacognitive knowledge (Armon-Lotem et al., 2015), their comprehension of the plot, and their ability to interpret and explain the perspectives and intentions of the protagonists (Curenton and Justice, 2004; Nippold et al., 2005; Heilmann et al., 2010).

Several studies compared macro and microstructure in the narrative productions of multilingual children. For example, Hipfner-Boucher et al. (2015) examined macrostructure and microstructure in the narrative retelling of 4–6 years old typically developing English Language Learners (ELL) with different home languages and compared those to monolingual English peers in Canada. All the ELL children had the same average exposure to English in their educational settings but had either a high or low exposure to English at home. With respect to microstructure, the low English-at-home group had significantly lower scores for sentence length, vocabulary, and grammaticality than the monolinguals and the high English-at-home group. However, both groups of ELL children produced story grammar of similar complexity to their monolingual peers. The differences in exposure to the dominant language at home influenced microstructure but not macrostructure in ELL early school-age children (Hipfner-Boucher et al., 2015). Narrative microstructure and macrostructure were also compared across languages in typically developing simultaneous Norwegian-Russian bilingual children (age 4–5 years) by Rodina (2017). Using the MAIN narrative assessment in tell mode, the study indicated that macrostructure was comparable across the two languages in both production and comprehension while microstructure was sensitive to language exposure. For the dominant language Norwegian, when compared to the monolingual peers, narratives of the bilingual children did not differ in either microstructure or macrostructure. However, for the minority language Russian when compared to the monolingual peers, narratives of the bilinguals differed in all microstructure measures while there was no difference in the macrostructure measure of story complexity. Similar results were obtained for balanced Polish-English bilinguals (age 5–7 years) living in the UK and attending education in English. Using the MAIN narrative assessment in both tell and retell mode Otwinowska et al. (2020) found that children's performance was comparable across the languages on all macrostructure measures while differences between productions in Polish and English were observed in basic lexical and syntactic measures which refer to microstructure.

Further comparison of microstructure and macrostructure in the narratives of bilingual children indicated that macrostructure might better discriminate between typically developing (TD) children and children with developmental language disorder (DLD). Narrative macrostructure was compared across TD children and children with language impairment (LI) in Dutch monolinguals and bilinguals (aged 5–6 years) by Boerma et al. (2016). Using the MAIN narrative assessment in the “tell after a different model story” mode of elicitation, the study indicated that macrostructure measures did not differ across monolingual and bilingual TD groups while, at the same time, macrostructure scores reliably differentiated between TD and LI groups in both monolinguals and bilinguals. Given that the current study is the first step toward the final aim of using MAIN Gaeilge (Irish) as a clinical language assessment tool, these findings point toward the advantage of macrostructure scores for this purpose.

The studies comparing microstructure and macrostructure for different language pairs indicated similar macrostructure across the languages in multilingual children. This is in line with the previous findings and theoretical assumptions suggesting that narrative macrostructure relies on children's cognitive development including general information processing skills such as working memory, attention, and executive function related skills of organization and planning (e.g., Berman and Slobin, 1994; Friend and Bates, 2014). It is further proposed that children may transfer domain general conceptual base across the languages resulting in equivalent narrative macrostructure for all their languages (Cummins, 1979; MacWhinney, 2005). Comparable macrostructure across languages of bilingual children makes narrative assessment a potentially useful tool for language assessment of Irish speakers. Because of the near-universal bilingualism and the variability in language exposure to Irish and English, a measure that is potentially equivalent across the two languages and at the same time can differentiate between TD children and children with DLD would be an ideal assessment tool for the population of Irish-English bilinguals.

Most studies comparing macrostructure across the languages of multilingual speakers focused on children. The study by Gagarina et al. (2019) compared narrative macrostructure production in German, Russian, and Swedish in monolingual adults. The aim of the study was to provide benchmark data from monolingual adults for story structure (sum of the core story elements G, A, O, and ISTs produced in the narrative) and story complexity (combinations of the core elements G, A, and O within each episode) and to compare those across the three languages. The MAIN Baby Goats and Baby Birds stories were used to elicit narratives employing the tell mode of elicitation. The story structure scores were similar across the languages, indicating that the elicitation pictures are cross-linguistically and cross-culturally robust. Adults did not show the ceiling effect and achieved relatively low scores for story structure with an average of 11–12 points out of a maximum of 17. When comparing the story structure scores for each story, Baby Goats' scores were slightly higher than Baby Birds' scores. This finding is consistent with the findings of Lindgren (2019) where higher scores for story comprehension were also found in Baby Goats' story. Narrative comprehension was not reported in this study. The findings provide important information about adults' production of narrative macrostructure and benchmark data for the MAIN story structure and story complexity in monolingual German, Russian, and Swedish speakers.

The current study contributes to the existing research by reporting data on narrative macrostructure production and comprehension by Irish-English bilingual adults and establishing a baseline for macrostructure measures in this population. Given the near universal bilingualism with English, it would be impossible to benchmark the narrative assessment scores in Irish speaking monolinguals. Establishing adult benchmarks for this population is necessary because of the rapid language change of the Irish language which is evident for each new generation of speakers. Furthermore, previous research employing narratives in Irish indicated that when telling a story to their children, adults used some morphosyntactic forms consistently and accurately while other forms they used either inconsistently or inaccurately. Crucially, the forms that parents used consistently and accurately were those that children acquired fully at an early age and used when retelling the same story (Müller et al., 2019; Antonijevic et al., 2020). Lead by those findings, we think that the first step towards creating children's norms for the MAIN in Irish and English is describing the story structure produced by adult Irish-English bilingual speakers, i.e., obtaining benchmarks to which children's narratives will be compared.

The research with multilingual children indicated that the macrostructure scores were similar across their languages (e.g., Hipfner-Boucher et al., 2015; Boerma et al., 2016; Rodina, 2017; Otwinowska et al., 2020). In addition, the macrostructure scores were similar in monolingual adults in different languages (Gagarina et al., 2019). Therefore, we expected the macrostructure scores in both production and comprehension to be similar across the endangered minority language Irish and the dominant language English in adult Irish-English bilinguals. This is the first step toward establishing the developmental trajectory for narrative production and comprehension in Irish-English bilingual speakers.

## Materials and methods

The study received full ethical approval from the College of Medicine, Nursing and Health Sciences Research Ethics Committee at the National University of Ireland Galway.

We report here on two studies, both using the MAIN in Irish (O Malley, 2019) and English (Gagarina et al., 2019). The studies used the same procedures and the same participants' inclusion and exclusion criteria and protocols. Study 1 used the Baby Birds and Baby Goats story pair and all participants were teachers in Irish medium education. Study 2 used Cat and Dog story pair and participants were recruited through social media.

### Participants

Participants who met the following criteria were invited to participate: healthy adults aged 20–60 years; regular speakers of both Irish and English; the household must have an Internet connection; the household must have a computer or an iPad with a webcam; participants must have or be willing to create a Zoom account; participants must have a quiet space available for the duration of the assessment. Participants could not participate in the study if they had any diagnosis of developmental or acquired language disorder, neurodegenerative, or other conditions that may impact speech, language, or cognitive abilities; or if they spoke daily any other languages in addition to Irish and English. This prevented the potential influence of another language on the participant's narrative while maintaining focus on macrostructural measures of Irish-English multilinguals. Prior to the narrative assessment, all participants completed an online demographic and language questionnaire The Language Experience and Proficiency Questionnaire (LEAP-Q; Marian et al., 2007) to establish their current language exposure and self-rated language proficiency in Irish and English (refer to Table 1).

**Table 1:** Demographic and language variables reported through LEAP-Q (Marian et al., 2007) for Study 1 and Study 2.

**Study**	**N**	**Age**	**Years in education**	**Current exposure to Irish**	**Current exposure to English**	**Age of Acquisition (AoA) Irish**	**Age of Acquisition (AoA) English**	**Proficiency in speaking Irish (0–10)**	**Proficiency in speaking English (0-10)**
1	20	38.2 (14.77)	17.85 (1.76)	38.9 (22.79)	60.85 (22.89)	4.9 (5.47)	0.75 (1.37)	8.75 (0.85)	9.55 (0.83)
2	10	36 (12.4)	18.2 (2.78)	39 (13.05)	60.5 (13)	7 (10.78)	2.5 (2.99)	9.1 (1.29)	8 (1.05)

Study 1 participants included 14 women and six men, aged between 23 and 54 years (*M* = 38.2, *SD* = 14.77). Participants reported a variation in the current language exposure (refer to Table 1). Three participants reported Irish as their first language (L1) and 17 reported Irish as their second language (L2). Study 2 participants included eight women and two men, aged between 22 and 59 years (*M* = 36, *SD* = 12.4). Irish was L1 of four participants while six participants had Irish as L2. One of the participants involved in the study was a Speech and Language Therapist by profession (TM5), however, they were not familiar with the MAIN. The current language exposure and age of acquisition for Irish as well as the self-rated proficiency for Irish and English are presented in Table 1. Detailed information about language history and proficiency in Irish and English is presented in the Supplementary Materials.

Participants in Study 1 and Study 2 were matched on age, years of education, current exposure to Irish and English, AoA for Irish and English, and self-rated proficiency in speaking Irish and English (refer to Table 1). Individual scores, means, and SDs for all language related variables reported through LEAP-Q (Marian et al., 2007) are presented in the Supplementary Materials.

### Materials and procedure

The MAIN (Gagarina et al., 2019) was the language assessment tool used to collect narrative data. The tool was developed to assess the narrative comprehension and production abilities of bilingual children 3–10 years of age. The MAIN includes four parallel stories (Cat, Dog, Baby Birds, and Baby Goats) each accompanied by a set of six pictures. All four stories include three distinct episodes, where each episode contains five elements: a goal, an attempt, an outcome, and two internal state terms positioned in the sequence as an initiating event and as a reaction. A goal (G) represents a statement of an idea of the protagonists to deal with the initiating event (e.g., “Mother bird wanted to catch worms”). An attempt (A) is an indication of action to obtain the goal (e.g., “Mother bird looked for food”); an Outcome (O) is the event following the attempt and is causally linked to it (e.g., “Mother fed the baby birds”). Internal state terms (ISTs) can be either an initiating event that sets the events of the story in motion (e.g., “Mother bird saw that the baby birds were hungry”) or a reaction that defines how the protagonist feels/thinks about the outcome (e.g., “Baby birds were happy/not hungry anymore”). Across the four stories the details related to the protagonists, background and foreground information, and content were controlled to allow for comparison between two languages or between elicitation modes. The MAIN is designed to use one of three elicitation modes: story tell, story retell, and story tell after listening to a different model story (Gagarina et al., 2012, 2019).

The theoretical approach underpinning the MAIN distinguishes two main aspects of macrostructure: story structure and story complexity. Story structure is a quantitative score reflecting the number of episodic elements produced in the narrative. It consists of a score for describing the story setting (a reference to time and place) and scores for elements present in each of the three episodes. Given that each story includes settings referring to time and place that are unique for all three episodes, and the three episodes that each contain G, A, and O as well as IST as an initiating event and IST as a reaction, the story structure score can reach a maximum of 17 (2 for settings and 5 for elements in each of the 3 episodes). While ISTs are included in the MAIN as a part of the macrostructure, they also form a bridge between narrative organization on a more general conceptual level and the linguistic encoding of this information at the lexical level. In addition to ISTs being a part of the story structure score, the MAIN includes a separate ISTs score referring to all instances of perceptual and physiological state terms, consciousness and emotion terms, mental verbs, and verbs of saying and telling.

Narrative comprehension in the MAIN is examined by a set of ten open-ended questions focusing on goals and ISTs, the elements of macrostructure that are not directly present in the pictures but must be inferred (Bohnacker and Gagarina, 2020). Three of the ten questions target G, one from each episode; Six questions target ISTs, three as an initiating event, and three as a reaction. One question focuses on inferencing and requires the participant to reason about the meaning of the whole story (Gagarina et al., 2012). Given that there are 10 questions that each can be awarded one point, the maximum comprehension score is 10 points (Gagarina et al., 2012, 2019).

The narrative assessment was conducted using the MAIN in English (Gagarina et al., 2019) and Irish (O Malley, 2019). The general administration procedure for the MAIN was followed. The order of the languages and the stories across languages were counterbalanced. An online moderated assessment was conducted *via* a professional Zoom account. A custom-made PowerPoint presentation embedding the 6 pictures for each story was used to conduct the narrative procedure and share the pictures with participants (Hamdani et al., 2021). Participants' responses were audio-recorded using the Audacity software. The story tell elicitation method was used. The assessment started with a short warm-up session in the same language as the assessment. After that, participants were shown three envelopes to choose a story. As per the MAIN protocol (Gagarina et al., 2012, 2019), this was done to create an illusion that the researcher did not know the story that the participant was about to tell. In the beginning, they saw all six pictures together to get acquainted with the whole story. Subsequently, they were asked to tell the story while seeing two pictures (representing one episode of the story) at a time. The researcher remained silent except for the general feedback signals. Following narrative production, participants were asked 10 comprehension questions. The assessment took approximately 15 min per participant. The whole procedure was repeated 1–2 weeks later in the other language. Procedures were identical for Study 1 and Study 2 except that the Baby Birds and Baby Goats story pair was used in Study1 and Cat and Dog story pair in Study 2. All researchers that were involved in data collection had been trained in telehealth administration as a part of their degree in speech and language therapy.

The same researcher conducted the assessment in both languages introducing an aspect of bilingual mode for participants (Grosjean, 1989). This is, however, unavoidable because of the near-universal multilingualism of Irish with English leading to all Irish speakers understanding that their communication partner is not only an Irish but also an English speaker.

### Data analyzes

All narratives and answers to comprehension questions were transcribed verbatim. The narratives were then analyzed for the two measures of macrostructure: story structure and ISTs. The answers to the comprehension questions were analyzed separately. Throughout data analysis, researchers referred to the scoring examples in the MAIN: Gaeilge (Irish) (O Malley, 2019) and the MAIN (Gagarina et al., 2019). No points were awarded for the repetition of the same elements.

Identical data analyses were conducted separately for Study 1 and Study 2. The following analyses concerned macrostructure measures: story structure, ISTs, and comprehension score of the MAIN in Irish and English. To address the aim of this study and examine whether there are differences in macrostructure scores across languages a general linear model was conducted with factors: language (Irish/English) and output type (production/comprehension) including story structure and story comprehension scores as dependant variables. Both scores were expressed as proportions, story structure out of 17 and story comprehension out of 10, to allow for direct comparison.

## Results

Prior to analyzing data related to the MAIN, participants' language experience obtained by LEAP-Q language questionnaire (Marian et al., 2007) was compared across Irish and English for Study 1 and Study 2. In Study 1, participants' current exposure was higher to English (*M* = 60.86, *SD* = 22.89) than to Irish (38.90, *SD* = 22.79) [*t*_(19)_ = 2.15, *p* = 0.05, *d* = 0.47] and their self-rated proficiency was also higher in English (*M* = 9.55, *SD* = 0.83) than Irish (*M* = 8.75, *SD* = 0.85) [*t*_(9)_ = 2.79, *p* = 0.01, *d* = 0.61]. AoA for English (*M* = 0.75, *SD* = 1.37) was lower than AoA for Irish (*M* = 4.9, *SD* = 5.47) [*t*_(19)_ = −3.237, *p* = 0.004, *d* = −0.724]. Similarly, in Study 2, participants' current exposure to English (*M* = 60.50, *SD* = 13.01) was higher than to Irish (39.50, *SD* = 13.01) [*t*_(9)_ = 2.55, *p* = 0.03, *d* = 0.77] and their self-rated proficiency was also higher in English (*M* = 9.1, *SD* = 1.29) than Irish (*M* = 8, *SD* = 1,05) [*t*_(9)_ = 2.4, *p* = 0.04, *d* = 0.73]. However, AoA for English (*M* = 2.5, *SD* = 2.99) was not significantly different from AoA for Irish (*M* = 7, *SD* = 10.78) [*t*_(9)_ = −1.12, *p* = 0.293, *d* = −0.35], which is most likely result of the high variability in AoA for Irish. Language variables reported for Study 1 and Study 2 are presented in Table 1 above.

### Comparison of story structure and story comprehension across Irish and English

**Figure 1:**
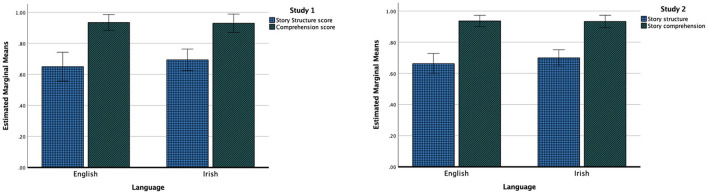
The MAIN story structure and story comprehension scores and standard deviations across Irish and English in Study 1 and Study 2.

In Study 1, the mean story structure score in Irish was 11.8 (*SD* = 2.53) and mean comprehension score in Irish was 9.30 (*SD* = 1.26); the mean English story structure score was 11.05 (*SD* = 3.38), and the mean comprehension score in English was of 9.35 (*SD* = 1.09). To be able to directly compare production and comprehension scores, the raw scores were transformed into proportions out of 17 for story structure and out of 10 for comprehension. A general linear model with factors language (Irish/English) and output type (production/comprehension) indicated no significant difference in the overall performance across languages [*F*_(1,19)_ = 0.615, *p* = 0.44, η^2^ = 0.031]. A significant overall difference was observed for output type [*F*_(1,19)_ = 60.85, *p* < 0.001, η^2^ = 0.76]. Participants performed significantly better in narrative comprehension than production, irrespective of the language. No significant interaction was found between language and output type [*F*_(1,19)_ = 1.04, *p* = 0.32, η^2^ = 0.05] indicating that the discrepancies between production and comprehension scores were the same in both languages (refer to Figure 1).

In Study 2, the overall mean story structure score in Irish was 11.7 (*SD* = 1.95), and the mean comprehension score in Irish was 9.4 (*SD* = 0.84); the mean story structure score for English was 12.10 (*SD* = 1.85), and mean comprehension score for English was 9.4 (*SD* = 0.52). Similar to Study 1, production and comprehension scores were subsequently transformed into proportions to enable their direct comparison.

A general linear model with factors language (Irish/English) and output type (production/comprehension) indicated no significant difference in the overall performance across languages [*F*_(1,9)_ = 0.29, *p* = 0.603, η^2^ = 0.031]. A significant difference was observed for output type [*F*_(1,19)_ = 192.64, *p* < 0.001, η^2^ = 0.96]. Participants performed significantly better in narrative comprehension than production, irrespective of the language. No significant interaction was found between language and output type [*F*_(1,19)_ = 0.159, *p* = 0.70, η^2^ = 0.017] indicating similar discrepancy between production and comprehension across the languages (refer to Figure 1).

Finally, combined analysis across Study 1 and Study 2 indicated no significant three-way interaction between the language, the type of production, and the story pair [*F*_(2,27)_ = 0.339, *p* = 0.715, η^2^ = 0.025] confirming that similar results were obtained across the two story-pairs Baby Birds and Baby Goats vs. Cat and Dog stories (refer to Figure 1).

### Internal state terms across Irish and English

**Figure 2:**
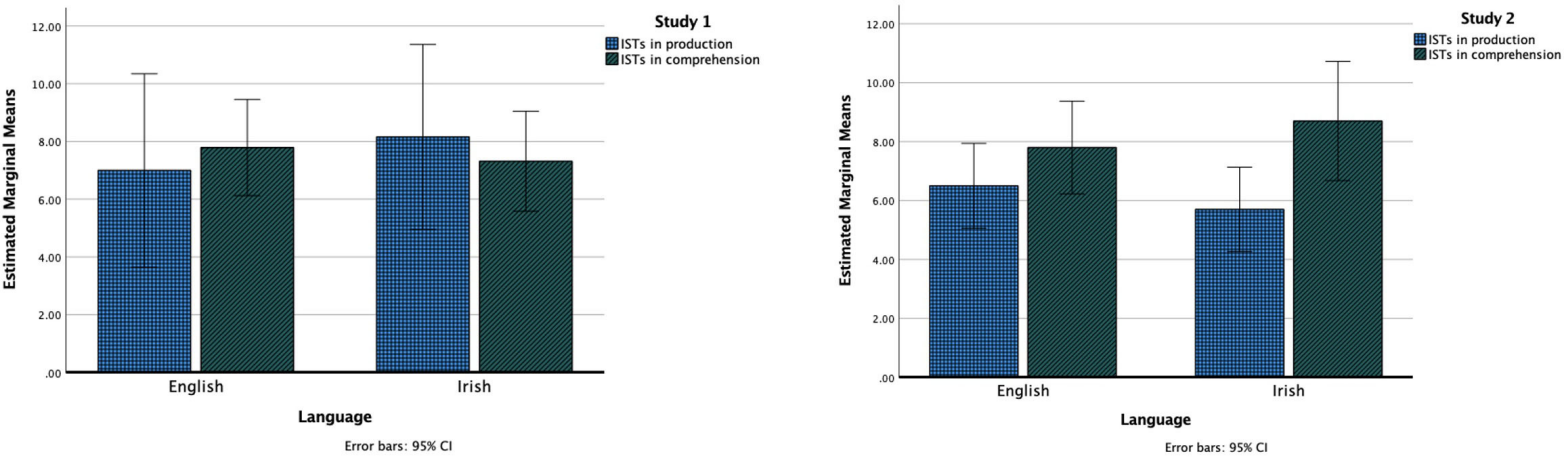
The internal state terms (ISTs) in production and comprehension across Irish and English in Study 1 and Study 2.

In Study 1 (Baby Birds and Baby Goats story pair), a general linear model with factors language (Irish/English) and output type (production/comprehension) indicated that there was neither significant difference in the number of ISTs across the languages [*F*_(1,19)_ = 0.229, *p* = 0.638, η^2^ = 0.013] nor the number of ISTs produced in comprehension vs. production [*F*_(1,19)_ = 0, *p* = 0.938, η^2^ = 0]. There was no significant interaction between the language and the output type [*F*_(1,19)_ = 1.685, *p* = 0.211, η^2^ = 0.086] (refer to Figure 2).

In Study 2 (Cat and Dog story pair), a general linear model with factors language (Irish/English) and output type (production/comprehension) indicated that there was no significant difference in the number of ISTs across the languages [*F*_(1,9)_ = 0.007, *p* = 0.935, η^2^ = 0.001]. However, a higher number of ISTs was produced in comprehension than in production [*F*_(1,9)_ = 6.12, *p* = 0.035, η^2^ = 0.405]. There was no significant interaction between the language and the output type [*F*_(1,9)_ = 0.159, *p* = 0.70, η^2^ = 0.017] (refer to Figure 2).

## Discussion

The aim of the current study was to establish measures of macrostructure in narrative production and comprehension for Irish-English adult bilinguals and to use those as a baseline for further comparison of narrative macrostructure in Irish-English bilingual children. There were two subsets of data, Study 1 employed the Baby Birds and Baby Goats story pair, and Study 2 employed Cat and Dog story pair from the Multilingual Assessment Instrument for Narratives (MAIN; Gagarina et al., 2012, 2019) to elicit narratives in the tell mode. Despite participants' different backgrounds (participants in Study 1 were teachers in Irish-immersion schools while participants in Study 2 had more diverse linguistic backgrounds), both groups indicated that in the recent past they had higher exposure to English than to Irish and also rated their proficiency in English to be higher than in Irish. This is likely the case due to the convergence of the languages, the universal bilingualism of the Irish language with English, and the global dominance of English. Similar results were previously reported in children in research by Nic Fhlannchadha and Hickey (2021), who concluded that young Irish-English bilinguals from Irish dominant homes are often the minority in Irish immersion education, reflecting that the majority of Irish-English bilinguals have an abundant exposure to English daily. This finding is also in line with the changes in the sociolinguistic landscape of Ireland that have been well documented (Ó Catháin, 2016).

### Macrostructure in comprehension and production across Irish and English

As expected on the basis of previous studies including children (e.g., Gagarina et al., 2015; Hipfner-Boucher et al., 2015; Boerma et al., 2016; Bohnacker, 2016; Gagarina, 2016; Rodina, 2017; Otwinowska et al., 2020) and monolingual adults (Gagarina et al., 2019), there was no difference in macrostructure scores across languages in either production or comprehension of narratives. These results were consistent across Study 1 and Study 2. A comparison of narrative macrostructure measures across production and comprehension indicated that overall comprehension scores were higher than production scores, but that this trend did not differ across the languages. In narrative production, mean story structure scores for both English and Irish were in the same range as observed by Gagarina et al. (2019). The mean story structure score was 11.8 for Irish and 11.05 for English concurring with those reported by Gagarina et al. (2019) being in the range of 11–12 points for monolingual German-, Russian-, and Swedish-speaking adults. These findings are encouraging because they suggest that story structure scores are stable across different languages and comparable between monolingual and multilingual adults. Therefore, the obtained scores can be used as a baseline to which children's narrative macrostructure scores will be compared in the future. In that context, it is important to notice that adults did not show the ceiling effect in either production or comprehension and that their narrative comprehension scores were higher than production scores. Similar to the current study, higher comprehension scores relative to production scores were also reported in previous studies (e.g., Bohnacker, 2016; Kapalková et al., 2016). One potential reason for this discrepancy was outlined by Bohnacker (2016) who observed that Goals and ISTs were frequently produced in response to the comprehension questions but rarely spontaneously produced in the narrative production. Goals and ISTs are not explicitly depicted in the elicitation pictures and the narrator needs to infer them from the story plot in narrative production. In narrative comprehension, these elements are specifically addressed in the questions and, therefore, attention is pointed toward them potentially making them easier to include in the answer. Closer inspection of the results of previous studies indicated that this gap between comprehension and production contracted with age (Bohnacker, 2016) and an increase in language exposure (e.g., Roch et al., 2016). In addition, higher story structure scores with more story grammar elements were observed in retell then tell mode (e.g., Kapalková et al., 2016; Roch et al., 2016), which could be a consequence of children hearing explicitly the elements that in the tell mode they would need to infer themselves. The ability to infer the elements not directly present in the pictures is related to the theory of mind and also understanding of the plot of the whole story (Gagarina, 2016). In this respect, both narrative production and comprehension require cognitive in addition to linguistics abilities. While cognitive and linguistic abilities are developing in children, and potentially leading to a reduction in the gap between story structure and story comprehension scores, it is important to know that adult Irish-English bilinguals still achieved higher scores in narrative comprehension than production. This data is an important benchmarking point for the comparison of narrative production and comprehension in Irish-English bilingual children. Narrative macrostructure has been shown to successfully differentiate between TD and DLD monolingual and multilingual children without disadvantaging multilingual TD children (Boerma et al., 2016), which is most likely due to its reliance on cognitive functions such as attention and the theory of mind (Blom and Boerma, 2016; Gagarina, 2016). Taking these findings together with the linguistic, cultural, and socioeconomic variability of Irish speakers leads us to believe that narrative macrostructure is the most optimal tool for language assessment of Irish-English bilingual children.

Finally, the fact that no significant difference in macrostructure measures across the languages and the types of output (comprehension vs. production) were observed for both Baby Birds and Baby Goats, as well as Cat and Dog story pairs, supports the original idea that the MAIN stories were created to have parallel macrostructure with the same number of episodes and the same episode structure (Gagarina et al., 2012, 2015).

### Internal state terms in production and comprehension across Irish and English

Similar to story structure and story comprehension scores, ISTs in narrative production and comprehension were compared across Irish and English. In Study 1 (Baby Birds and Baby Goats stories) similar number of ISTs was observed in production and comprehension, and also across the languages. However, in Study 2 (Cat and Dog stories) a higher number of ISTs was observed in comprehension relative to production and this was the case in Irish and English. While this could indicate differences in the story pairs with respect to the elicitation of ISTs, it is important to note that Study 2 had a smaller number of participants whose backgrounds differed from that of the participants in Study 1. While participants in Study 1 were recruited through the Irish-medium schools in English dominant areas where they worked as teachers, participants in Study 2 were recruited through social media and had more diverse backgrounds so the discrepancy in the number of ISTs in production and comprehension could be driven by participants' characteristics. A higher number of ISTs in narrative comprehension relative to production has been observed by Bohnacker (2016) in Swedish-English bilingual children aged 5–7 years. In the study by Bohnacker, ISTs as initiating events and ISTs as reactions were produced in the majority of cases in comprehension questions, however, they were rarely spontaneously produced in narrative production. Furthermore, the number of ISTs as initiating events increased in the narrative production from age 5 to 7 years, but this was not true for the number of ISTs as reactions (Bohnacker, 2016). ISTs as reactions involve understanding the complete story plot, referring to the theory of mind, and inferring how the characters in the story might feel. Therefore, the findings observed in the current study could be pointing toward the difficulty to infer characters' mental states and including them in the narrative production. A similar type of difficulty was observed in a study that examined another minority language, Gaelic, with respect to inference in reading comprehension. Dickson et al. (2021) found that primary school children in Gaelic-medium education who had English as their dominant language struggled to answer questions requiring them to infer information from a paragraph they read. This difficulty, however, was not observed for both languages of the English-Gaelic bilinguals, which is different from the current study. Discussing the nature of ISTs, Gagarina (2016) suggested that ISTs are much more dependent on lexical knowledge than other macrostructure components. Therefore, the difference in the number of ISTs in production and comprehension observed in the current study could be a result of the potential discrepancy between receptive and expressive vocabulary in both languages for this group of participants. To understand whether the observed pattern of results reflects differences in the two sets of stories or whether it is a result of the characteristics of the participants, all four stories would need to be compared across the same group of Irish-English bilinguals, which will be the aim of future studies.

### Code-switching

Narrative assessment is particularly suited for multilinguals because it allows for observation of the phenomena specific to language production in multilinguals such as code-switching (Gagarina et al., 2015). Frequent code-switching with English is a significant characteristic of modern Irish (Ó Catháin, 2016). Code-switching was evident during Irish narrative production and comprehension in the current study. English words were frequently used while beginning the Irish narrative production, and included “OK, so,” “OK,” and “So.” Despite “OK” having a direct Irish translation, “*ceart go leor.”* “OK, so” and “so” do not have direct Irish translations, indicating a lexical gap (Ní Laoire, 2016). Participants may have used these words and phrases to emphasize a point (Ní Laoire, 2016), e.g., at the beginning of the episode. Similarly, numerous participants used code-switching to English to emphasize the end of a sentence or episode, using phrases such as “*Sin alright?*” or “Is that alright?,” “*Sin é really”* or “That's it really” and “Em yeah.” This type of code-switching indicates metalinguistic awareness where a different language is used to emphasize the change in topic. Some participants used code-switching when they were unaware of the correct Irish expression e.g., using “nest” instead of *nead* and “the cat ran away” instead of *rith an cat leis*. However, due to the bilingual approach of the MAIN Gaeilge (Irish) (O'Malley and Antonijevic, 2020), these responses were marked as correct. Interestingly, participants used code-switching as a form of linking sentences throughout narrative productions, despite those words existing in Irish. Those conjunctions included “yeah,” “you know,” “because,” “and then,” “really,” “either” and “alright,” “is it?” and “is that what you mean?.” We suspect that this is likely a result of the almost universal bilingualism of the Irish sociolinguistic context' (O'Malley and Antonijevic, 2020, p. 127), and participants being in a bilingual mode (Grosjean, 1989) as well as knowing that the researcher is also multilingual and will understand their responses in both Irish and English. One participant (CK4) also used the verb “scalaíonn” when describing the cat climbing up the tree. As this is not a verb in Irish, this may be an example of *Béarlachas* or “Englishism,” which describes the contact between Irish and English (Ní Laoire, 2016, p. 101). The features of code-switching outlined above are aligned with those described by Ní Laoire (2016), and reports by Ó Catháin (2016) who described the younger generations of Irish speakers using Irish differently from that of the previous generations.

## Online administration of the MAIN

All three researchers who participated in data collection had previous experience with telehealth. They found the administration of the MAIN online to be straightforward. It was helped by the clear instructions, a slideshow of pictorial stimuli, and clear visuals for comprehension questions (Hamdani et al., 2021). The online platform allowed for good rapport building at the beginning of the assessment. Online administration improved time management and allowed for flexibility in arranging assessment times and dealing with cancellations. However, because the home environment being more intimate than a research lab or a clinic, the researchers had the impression that some participants were more guarded. Particularly relevant for Irish settings was that conducting the assessment online allowed for greater geographical reach in recruiting participants. The highest density of Irish speakers is in rural and sometimes remote areas of Ireland. The in-person assessment would hinder their participation because either researchers or participants would have to travel to the place of assessment and in this way significantly increase the time and the cost involved. On the other hand, during in-person assessment researcher has more control over the environment with no risk of internet connection breakdown, difficulties with sound, or participants not having a quiet place in their home. With respect to communication, it was sometimes difficult to read facial expressions or body language. Communication was also sometimes impaired by poor internet connection which could cause overlap when giving instructions and asking comprehension questions. Future studies could work on minimizing the downsides of online assessment given that this mode of assessment has good potential to be used in clinical settings (Peña and Sutherland, 2022).

### Limitations

This study has several limitations. The smaller sample size in Study 2 is a limitation and future research should include a larger number of participants. A further limitation is that the MAIN was originally created for an in-person assessment. As a result of the COVID-19 pandemic, the MAIN was subsequently adapted for online use (Hamdani et al., 2021). The online administration of the MAIN was employed in the study by Pratt et al. (2022). They noted during the data collection that participants did not always clearly see what was going on in the pictures and that the size of the pictures needed to be increased. This current study used the original PowerPoint slides (Hamdani et al., 2021) and the picture size was not increased. This problem became clear because on re-examination of the picture stimuli during the comprehension questions, the story was clarified for some participants. Multiple individuals incorrectly described the first episode of the Baby Goats story as the goat “cooling down” or “swimming.” However, it became clear to the same participants during the comprehension task that the baby goat was in fact drowning, with one participant (CK8) exclaiming “oh actually, she might be looking for ah help.” Not seeing the pictures clearly may have impacted the participants' ability to produce full GAO sequences, and may have resulted in higher mean comprehension scores in comparison to the mean story structure scores. The size of the pictures for online administration should be adjusted in the future to enable participants to better view the details.

## Conclusion

The current study aimed to compare macrostructure measures in the MAIN stories (Gagarina et al., 2012, 2019) across output type (production vs. comprehension) in the two languages of Irish-English multilinguals. This study also aimed to establish a baseline for macrostructural measures in Irish and English using the MAIN and MAIN Gaeilge (Irish) (O Malley, 2019) that can be used in future research as well as in clinical settings.

The similarity in macrostructure measures that were obtained across languages during production and comprehension of narratives indicated that differences in language exposure, AoA, and self-rated proficiency between Irish and English did not influence measures of macrostructure in either production or comprehension. Therefore, the results of the current study suggest that the MAIN macrostucture measures are not sensitive to linguistic variability, which is the characteristic of Irish-English multilinguals throughout Ireland and is due to the ever-increasing use of the majority language English and the decreasing use of the minority language Irish. This implies that the MAIN is an optimal language assessment tool for Irish-English bilingual children. The mean story structure and story comprehension scores observed in this study may be cautiously used as a baseline for measures of macrostructure among Irish-English multilinguals. Future studies should focus on using the MAIN (Gagarina et al., 2012, 2019) and MAIN Gaeilge (Irish) (O Malley, 2019) to assess Irish-English bilingual children and determine the developmental trajectories for measures of the macrostructure. The final aim is to provide a valuable tool for language assessment of Irish-English multilingual children in clinical settings, the tool that can overcome challenges of language assessment of a fast changing, endangered minority language Irish. In addition, having an option of online assessment would enable clinicians to reach children across Ireland that are in need of language assessment. The current study is one of the first steps toward that goal.

## Data availability statement

The raw data supporting the conclusions of this article will be made available by the authors, without undue reservation.

## Ethics statement

The studies involving human participants were reviewed and approved by Research Ethics Committee of the College of Medicine, Nursing and Health Sciences, National University of Ireland Galway. The patients/participants provided their written informed consent to participate in this study.

## Author contributions

SA designed the research, contributed to the analyses, and writing of the manuscript. SC, CK, and TN contributed to the data collection, data analyses, and writing of the manuscript. All authors contributed to the article and approved the submitted version.

## Conflict of interest

The authors declare that the research was conducted in the absence of any commercial or financial relationships that could be construed as a potential conflict of interest.

## Publisher's note

All claims expressed in this article are solely those of the authors and do not necessarily represent those of their affiliated organizations, or those of the publisher, the editors and the reviewers. Any product that may be evaluated in this article, or claim that may be made by its manufacturer, is not guaranteed or endorsed by the publisher.
