# Supplementary material for: Online assessment of narrative macrostructure in adult Irish-English multilinguals
Source: Front Psychol. 2022 Jul 26;13:916214. doi: 10.3389/fpsyg.2022.916214 (PMC9374173; doi:10.3389/fpsyg.2022.916214)
Supplement: Supplementary file 1 [file Table_1.DOCX]

*Supplementary material; Table – Irish and English language variables gathered from LEAP-Q and used during analysis of macrostructure measures.*

|  | Exposure to Irish (%) | Age acquired Irish (years) | Time in English & Irish speaking country (years) | Time in Irish speaking family (years) | Time in Irish speaking home/work environment (years) | Proficiency speaking Irish  (scale 0-10) | Proficiency understanding Irish  (scale 0-10) | Proficiency reading Irish  (scale 0-10) |
| --- | --- | --- | --- | --- | --- | --- | --- | --- |
| CK1 | 40 | 1 | 48 | 48 | 11 | 8 | 9 | 9 |
| CK2 | 25 | 26 | 3 | 0 | 1 | 8 | 8 | 8 |
| CK3 | 80 | 8 | 56 | 32 | 32 | 9 | 9 | 9 |
| CK4 | 20 | 3 | 22 | 23 | 8 | 7 | 8 | 8 |
| CK5 | 3 | 4 | 20 | 0 | 8 | 8 | 9 | 8 |
| CK6 | 5 | 3 | 52 | 52 | 40 | 8 | 8 | 8 |
| CK7 | 95 | 10 | 53 | 40 | 47 | 9 | 10 | 10 |
| CK8 | 20 | 4 | 53 | 0 | 17 | 9 | 10 | 9 |
| CK9 | 25 | 3 | 22 | 19 | 14 | 8 | 9 | 10 |
| CK10 | 10 | 4 | 52 | 4 | 48 | 10 | 10 | 10 |
| SC1 | 50 | 4 | 30 | 0 | 23 | 10 | 9 | 9 |
| SC2 | 40 | 4 | 23 | 0 | 14 | 9 | 8 | 8 |
| SC3 | 40 | 5 | 22 | 0 | 14 | 9 | 9 | 9 |
| SC4 | 40 | 4 | 22 | 0 | 14 | 8 | 8 | 8 |
| SC5 | 40 | 4 | 59 | 0 | 15 | 8 | 8 | 8 |
| SC6 | 45 | 3 | 26 | 0 | 23 | 9 | 8 | 9 |
| SC7 | 50 | 0 | 60 | 60 | 20 | 10 | 9 | 9 |
| SC8 | 50 | 5 | 38 | 0 | 26 | 9 | 9 | 9 |
| SC9 | 50 | 3 | 27 | 0 | 21 | 9 | 8 | 9 |
| SC10 | 50 | 0 | 34 | 34 | 28 | 10 | 10 | 10 |
| Mean | 38.9 | 4.9 | 36.1 | 15.6 | 21.2 | 8.75 | 8.8 | 8.85 |
| Median | 40 | 4 | 32 | 0 | 18.5 | 9 | 9 | 9 |
| SD | 22.8 | 5.47 | 16.64 | 21.06 | 12.68 | 0.85 | 0.77 | 0.75 |
| Range | 92 | 26 | 57 | 60 | 47 | 3 | 2 | 2 |
|  | **Exposure to English (%)** | **Age acquired English (years)** | **Education**  **(years)** | **Time in English speaking family (years)** | **Time in English speaking home/work environment (years)** | **Proficiency speaking English (scale 0-10)** | **Proficiency understanding English**  **(scale 0-10)** | **Proficiency reading English**  **(scale 0-10)** |
| CK1 | 60 | 1 | 16 | 48 | 48 | 9 | 9 | 9 |
| CK2 | 75 | 0 | 21 | 44 | 44 | 10 | 10 | 10 |
| CK3 | 20 | 5 | 22 | 20 | 1 | 9 | 9 | 9 |
| CK4 | 80 | 1 | 18 | 22 | 10 | 10 | 10 | 10 |
| CK5 | 97 | 0 | 15 | 20 | 8 | 10 | 10 | 10 |
| CK6 | 95 | 1 | 19 | 52 | 48 | 10 | 10 | 10 |
| CK7 | 5 | 1 | 19 | 12 | 4 | 7 | 8 | 9 |
| CK8 | 80 | 0 | 15 | 53 | 53 | 10 | 10 | 10 |
| CK9 | 75 | 1 | 18 | 22 | 3 | 10 | 10 | 10 |
| CK10 | 90 | 0 | 16 | 52 | 48 | 10 | 10 | 10 |
| SC1 | 50 | 0 | 19 | 30 | 4 | 10 | 10 | 10 |
| SC2 | 60 | 0 | 18 | 23 | 5 | 10 | 9 | 9 |
| SC3 | 60 | 0 | 18 | 22 | 4 | 10 | 10 | 10 |
| SC4 | 60 | 0 | 17 | 22 | 4 | 9 | 9 | 9 |
| SC5 | 60 | 0 | 16 | 59 | 44 | 10 | 10 | 10 |
| SC6 | 50 | 1 | 18 | 26 | 22 | 9 | 10 | 9 |
| SC7 | 50 | 0 | 18 | 60 | 40 | 10 | 10 | 10 |
| SC8 | 50 | 0 | 18 | 38 | 12 | 10 | 10 | 10 |
| SC9 | 50 | 0 | 18 | 27 | 6 | 10 | 10 | 10 |
| SC10 | 50 | 4 | 18 | 24 | 1 | 8 | 9 | 8 |
| Mean | 60.85 | 0.75 | 17.85 | 34.30 | 20.45 | 9.55 | 9.65 | 9.6 |
| Median | 60 | 0 | 18 | 28.5 | 9 | 10 | 10 | 10 |
| SD | 22.89 | 1.37 | 1.76 | 15.08 | 20.21 | 0.83 | 0.59 | 0.6 |
| Range | 92 | 5 | 7 | 48 | 52 | 3 | 2 | 2 |
